# CD4^+^ T Cells of Myasthenia Gravis Patients Are Characterized by Increased IL-21, IL-4, and IL-17A Productions and Higher Presence of PD-1 and ICOS

**DOI:** 10.3389/fimmu.2020.00809

**Published:** 2020-05-19

**Authors:** Merve Çebi, Hacer Durmus, Fikret Aysal, Berker Özkan, Gizem Engin Gül, Arman Çakar, Mehmet Hocaoglu, Metin Mercan, Sibel P. Yentür, Melih Tütüncü, Vildan Yayla, Onur Akan, Öner Dogan, Yeşim Parman, Güher Saruhan-Direskeneli

**Affiliations:** ^1^Department of Physiology, Istanbul Medical Faculty, Istanbul University, Istanbul, Turkey; ^2^Department of Neurology, Istanbul Medical Faculty, Istanbul University, Istanbul, Turkey; ^3^Department of Neurology, Medipol University, Istanbul, Turkey; ^4^Department of Thoracic Surgery, Istanbul Medical Faculty, Istanbul University, Istanbul, Turkey; ^5^Okmeydani State Hospital, Istanbul, Turkey; ^6^Bakirköy Sadi Konuk State Hospital, Istanbul, Turkey; ^7^Department of Neurology, Cerrahpaşa Medical Faculty, Istanbul University Cerrahpaşa, Istanbul, Turkey; ^8^Department of Pathology, Istanbul Medical Faculty, Istanbul University, Istanbul, Turkey

**Keywords:** T follicular helper cells, PD-1, ICOS, IL-21, IL-4, IL-17, CXCR5, myasthenia gravis

## Abstract

Myasthenia gravis (MG) is an autoimmune disease mediated by autoantibodies predominantly against the acetylcholine receptor (AChR). Specific T cell subsets are required for long-term antibody responses, and cytokines secreted mainly from CD4^+^ T cells regulate B cell antibody production. The aim of this study was to assess the differences in the cytokine expressions of CD4^+^ T cells in MG patients with AChR antibodies (AChR-MG) and the effect of immunosuppressive (IS) therapy on cytokine activity and to test these findings also in MG patients without detectable antibodies (SN-MG). Clinically diagnosed AChR-MG and SN-MG patients were included. The AChR-MG patients were grouped as IS-positive and -negative and compared with age- and sex-matched healthy controls. Peripheral blood mononuclear cells were used for *ex vivo* intracellular cytokine production, and subsets of CD4^+^ T cells and circulating follicular helper T (cTfh) cells were detected phenotypically by the expression of the chemokine and the costimulatory receptors. Thymocytes obtained from patients who had thymectomy were also analyzed. IL-21, IL-4, IL-10, and IL-17A productions in CD4^+^ T cells were increased in AChR-MG compared to those in healthy controls. IS treatment enhanced IL-10 and reduced IFN-γ production in AChR-MG patients compared to those in IS-negative patients. Increased IL-21 and IL-4 productions were also demonstrated in SN-MG patients. Among CD4^+^ T cells, Th17 cells were increased in both disease subgroups. Treatment induced higher proportions of Th2 cells in AChR-MG patients. Both CXCR5^+^ and CXCR5^−^ CD4^+^ T cells expressed higher programmed cell death protein 1 (PD-1) and inducible costimulatory (ICOS) in AChR-MG and SN-MG groups, mostly irrespective of the treatment. Based on chemokine receptors on CXCR5^+^PD-1^+^ in CD4^+^ T (cTfh) cells, in AChR-MG patients without treatment, the proportions of Tfh17 cells were higher than those in the treated group, whereas the Tfh1 cells were decreased compared with those in the controls. The relevance of CXCR5 and PD-1 in the pathogenesis of AChR-MG was also suggested by the increased presence of these molecules on mature CD4 single-positive thymocytes from the thymic samples. The study provides further evidence for the importance of IL-21, IL-17A, IL-4, and IL-10 in AChR-MG. Disease-related CD4^+^T cells are identified mainly as PD-1^+^ or ICOS^+^ with or without CXCR5, resembling cTfh cells in the circulation or probably in the thymus. AChR-MG and SN-MG seem to have some similar characteristics. IS treatment has distinctive effects on cytokine expression.

## Introduction

Myasthenia gravis (MG) is an autoimmune disease characterized by fatigable muscle weakness caused by pathologic autoantibodies. The majority of MG patients (80–85%) have autoantibodies against acetylcholine receptor (AChR). Autoantibodies against muscle-specific kinase (MuSK) are present in a smaller subgroup of patients (1, 2). A small proportion of MG patients [10–15%, classified as seronegative MG (SN-MG)] do not have detectable autoantibodies against these antigens. A clinical comparison between AChR-MG, MuSK-MG, and SN-MG has revealed that the SN-MG patients were closer to the AChR-MG patients rather than to the MuSK-MG patients (3). Several findings in SN-MG support the possible role of autoantibodies related to AChR which can be detected by more sensitive assays in patients considered to be seronegative (4–6).

Thymus, the organ for development of self-tolerance, reveals different abnormalities in MG subtypes. In early-onset patients, the thymus is typically enlarged and contains many follicular germinal centers with T and B cells similar to those seen in the lymph nodes (7). Thymic hyperplasia with follicular structures frequently accompanies AChR-MG and is also detected in some SN-MG patients (8). However, distinct gene signatures in thymic samples from AChR-MG and SN-MG have also been demonstrated, underlining the different mechanisms of these disease subtypes (9).

T follicular helper (Tfh) cells, as a specialized subset of CD4^+^ T lymphocytes, are necessary for the generation of germinal centers (GC) in secondary lymphoid organs (10, 11). These cells are major producers of IL-21 which promotes B cell differentiation, antibody production, and Ig isotype switching, resulting in long-lasting antibody responses (12, 13). Tfh cells express transcription factor Bcl-6 and are characterized by their surface expression of C-X-C chemokine receptor type 5 (CXCR5), inducible costimulatory (ICOS), and programmed cell death protein 1 (PD-1) (14). Some studies have identified Tfh cells as total CXCR5^+^CD4^+^ T cells, while others have used subsets of CD4^+^ T cells such as CXCR5^+^ICOS^+^, CXCR5^+^PD-1^+^, CXCR5^+^ICOS^+^PD-1^+^, or CXCR5^+^IL-21^+^ (15). A circulating Tfh (cTfh) population has been described, which also expresses CXCR5, PD-1, and ICOS and can help B cell differentiation into plasma cells *via* IL-21 secretion (16).

An increase in the frequencies of cTfh populations is associated with several autoimmune diseases including rheumatoid arthritis (RA) (17), systemic lupus erythematosus (SLE) (18), and systemic sclerosis (SSc) (19). Recently, a pathologically expanded population of CXCR5^−^PD-1^hi^CD4^+^ T cells called T peripheral helper (Tph) cells has been identified in the synovium of patients with RA, which could also promote plasma cell differentiation (20). CXCR5^−^PD-1^+^CD4^+^T cell numbers and frequencies in blood positively correlated with plasma cells in patients with SSc (19). Both CXCR5^−^PD-1^+^CD4^+^ and CXCR5^+^PD-1^+^CD4^+^ T cells have been shown to produce high IL-21 (21). These findings implicate that the presence of the PD-1 molecule seems to be more effective than the presence of the CXCR5 molecule in antibody production.

Increased frequencies of ICOS^hi^ or PD-1^hi^CXCR5^+^CD4^+^ T cells with correlating serum AChR antibodies were reported in MG (22). A significant enrichment of activated (ICOS^+^) cTfh (CD4^+^CXCR5^+^PD-1^+^) cells has been assigned to Tfh subsets, namely, Tfh1 and Tfh17 cells, and these subsets were identified as the major source for IL-21 in generalized MG patients (22, 23). A demonstration of Tfh and B cells co-localized within the ectopic GC in MG thymus has also suggested the putative existence of intrathymic Tfh/B cell interaction playing a key role in this disease (24).

The pathogenesis of MG is generally characterized by various cytokines (25). Cytokine measurements in the sera revealed conflicting results: higher levels of IL-21 and IL-6 (23) or no significant increase in IL-21, IL-4, and IL-6 levels in AChR-MG patients (26) has been reported. Similarly, increased IL-17 in the sera of MG patients (27, 28) and similar levels among healthy controls (HC) in the sera or the culture supernatants of AChR-MG patients were demonstrated (26, 29).

A study measuring cytokine production from AChR-specific single-cell clones of MG patients demonstrated the co-expression of IFN-γ, IL-17, and GM-CSF, but not IL-10 (30). The heterogeneity of the disease and the effect of IS treatment may have caused these discrepancies between the studies which need clarification.

The anti-inflammatory properties of IS treatment result from the downregulation of pro-inflammatory or upregulation of anti-inflammatory genes. Several studies have demonstrated that glucocorticoids enhance the concentration of IL-10 in cultures of peripheral blood mononuclear cells (PBMCs) from HC *in vitro* (31, 32). In addition, the IL-10 levels were increased in the sera of MG patients who received IS treatment, and the IFN-γ levels of these patients were also lower than those of the controls (26). On the other hand, some studies have shown the effects of glucocorticoids on the Tfh population. Glucocorticoids decreased the CXCR5^+^PD-1^+^CD4^+^ Tfh cell population in SLE (33). In another study, the Tfh cells and the plasmablasts were decreased after steroid therapy in patients with IgG4-related disease (34).

IL-21, IL-4, IL-17A, IL-10, and IFN-γ were previously shown to be involved in the pathogenesis of MG (23, 26, 35). Based also on previous data, we aimed to further characterize the CD4^+^ T cells producing these relevant cytokines in AChR-MG and SN-MG. Among the CD4^+^ T cells, the role of cTfh cells or their functional molecules, PD-1 and ICOS, was investigated for differential contribution to disease development as well as to treatment responses in this study. The cytokines and their producer cells were compared *ex vivo* in the IS treatment groups. Moreover, changes in the thymic tissue cells of AChR-MG patients parallel with the peripheral T cells were also investigated.

## Materials and Methods

### MG Patient Blood Samples

Blood samples from 95 MG patients and 64 HC were included in this study (Table 1). The diagnosis of MG was based on clinical presentation, electrophysiologic examination, and presence of AChR antibodies. Of the 73 AChR antibody-positive patients, 34 had early onset (<50 years, AChR-EO,) and 39 had late onset (≥50 years, AChR-LO). In the AChR-MG group, 31 patients were on IS drugs (steroid alone or steroid plus azathioprine, ISP group) at the time of blood sampling, whereas 42 patients were not receiving IS treatment (ISN). In this group, 24 patients were previously thymectomized and the pathological classifications are shown in Table 1. Additional 22 patients without AChR and MuSK antibodies were included in the SN-MG group, eight (36%) of whom were thymectomized and were receiving treatment at the time of blood sampling. Thymoma-associated MG cases were not included in this study. In the AChR-MG group, seven patients (women/men: 2/5, AChR-EO/AChR-LO: 2/5), who were initially not on IS treatment, were followed for 6 months after starting the treatment and tested again while under treatment. The age and gender distributions of the patients and HC were balanced to be not different from each other. AChR and MuSK antibodies were measured by radioimmunoassay (DLD Diagnostika GmBH, Germany) and ELISA (Euroimmune, Germany) with commercial kits.

**Table 1:** Characteristics of the patients included in the study for peripheral blood cell evaluations.

**Characteristics**	**AChR-MG patients (*n* = 73)**	**SN-MG patients (*n* = 22)**	**HC (*n* = 64)**
Median age (years)	53 (14–79)	48 (18–73)	47 (27–76)
Women (%)	40 (55)	15 (68)	34 (53)
Disease onset (EO/LO)	34/39	15/7	–
Immunosuppressive treatment (%)	31 (43)	8 (36)	–
Thymectomy (%)	24 (33)	8 (36)	–
Hyperplasia	18	2	
Involution	6	6	

*AChR-MG, myasthenia gravis patients with AChR antibodies; SN-MG, myasthenia gravis patients without detectable antibodies; EO, early onset; LO, late onset*.

This study was approved by the Ethical Review Board of the Istanbul Medical Faculty. Peripheral blood was obtained from the donors after acquiring informed consent.

### Thymic Tissue Cells

Thymic tissue samples were obtained separately from the blood samples during therapeutic thymectomy in MG patients (Table 2). From only five patients, peripheral blood was taken simultaneously at the time of thymectomy. According to the thymic pathology, 33 (89.2%) of the AChR-MG patients had thymic hyperplasia and only four patients (10.8%) had involution. The isolated tissue cells from non-myasthenic patients with different ages (0–51) and who were undergoing corrective cardiovascular surgery were evaluated as controls (Con). The samples were taken from the parenchymal parts of the specimens under the guidance of a pathologist and were processed within a few hours. Cell suspensions from the thymic samples were obtained by mechanical manipulation, filtration through a cell strainer (100 μm, Life Sciences), and separation using the density gradient method to obtain the mononuclear cells. The final thymocyte suspensions were washed twice in staining buffer (phosphate-buffered solution containing 1% bovine serum albumin, SB). The analysis of molecules on freshly isolated cells was performed using three-color immunofluorescence by flow cytometry as described below.

**Table 2:** Features of the donors included in the study for thymic samples.

	**M (%)**	**W (%)**	**Total**	**Median age**	**ISP (%)**	**ISN (%)**
AChR-MG	1 (3)	36 (97)	37	26 (9–51)	16 (43.2)	21 (56.8)
Hyperplasia			33			
Involution			4			
Con	7 (36)	13 (64)	20	8 (0–51)		

*The myasthenia gravis patients were divided into immunosuppressive treatment positive (ISP) and negative (ISN) groups*.

*M, men; W, women; AChR-MG, myasthenia gravis patients with AChR antibodies; Con, thymic tissues from patients without myasthenia gravis*.

### Phenotypic Analyses of Peripheral Blood and Thymic Samples

PBMCs were separated by a gradient centrifugation procedure on a lymphocyte separation medium (Secoll Separation Media, Mannheim, Germany). After separation, freshly isolated cells were stained with the following fluorophore-conjugated antibodies: CD4 APC-Cy7 (clone: RFT-4g), ICOS PE-Cy7 (clone: ISA-3, eBioscience, ThermoFisher), CXCR5 FITC (CD185, clone: REA303), CXCR3 APC (CD183, clone: REA232), PD-1 PE-Vio 770 (clone: PD-1.3.1.3, Miltenyi Biotec), CD45RA PE-Cy5 (clone: HI100, BioLegend), and CCR6 PE (CD196, clone: 11A9, BD Bioscience).

The freshly isolated thymocytes and the PBMCs obtained at the time of thymectomy were stained with the following fluorochrome-conjugated antibodies: CD4 FITC (clone: RPA-T4, Beckman Coulter), CD8 APC (clone: RPA-T8), CXCR3 eFluor660 (CD183, clone: CEW33D, eBioscience), CCR6 PE (CD196, clone: 11A9), PD-1 PE (CD279, clone: MIH4, BD Bioscience), ICOS PE (clone: C398.4A, BioLegend), and CXCR5 PE (CD185, clone: 51505, R&D Systems). The samples were analyzed on an Attune Flow Cytometry (Thermofisher, USA). Fluorescence minus one control, which contains all flurochromes in a panel except for the target markers measured, was used to identify and to gate the cells.

### Intracellular Staining

Freshly isolated PBMCs were seeded in 48-well plates at a final concentration of 2 × 10^6^ cells/ml in complete RPMI1640 medium supplemented with 2 mM L-glutamine, 100 IU/100 mg/ml penicillin/streptomycin (Sigma), and 10% fetal bovine serum (Gibco) and were stimulated by the cell stimulation cocktail (500X) containing phorbol 12-myristate 13-acetate and ionomycin (eBioscience, ThermoFisher) for 4 h at 37°C. After washing with SB, the cells were surface-stained with anti-CD4 antibody (clone: RPA-T4, BioLegend) for 20 min on ice. Then, the cells were permeabilized and fixed with a fixation/permeabilization solution (BD Bioscience) according to the manufacturer's instructions. The cells were stained with IL-21 PE (clone: 3A3-N2), IL-4 Alexa Fluor 488 (clone: 8D4-8), IL-17A PE (clone: eBio64), IFN-γ Alexa Fluor 488 (clone: 4S.B3), or IL-10 Alexa Fluor 488 (clone: JES3-9D7, eBiosciences Thermofisher) for 30 min on ice. The results were acquired by flow cytometry as discussed above.

### Statistical Analysis

In the statistical analysis measurements, Shapiro–Wilk test for normality was applied. As the distributions of variables were not normal in the sample populations, non-parametric tests (Kruskal–Wallis and Mann–Whitney U) were used with SPSS version 21. In the comparison of thymic samples, covariance analysis (ANCOVA) was applied for controlling the age differences between patients and controls. The data were presented as median values with interquartile ranges in the figures. A *p* < 0.05 was considered as statistically significant.

## Results

### Elevated IL-21, IL-4, IL-17A, and IL-10 Production in MG Patients

**Figure 1:**
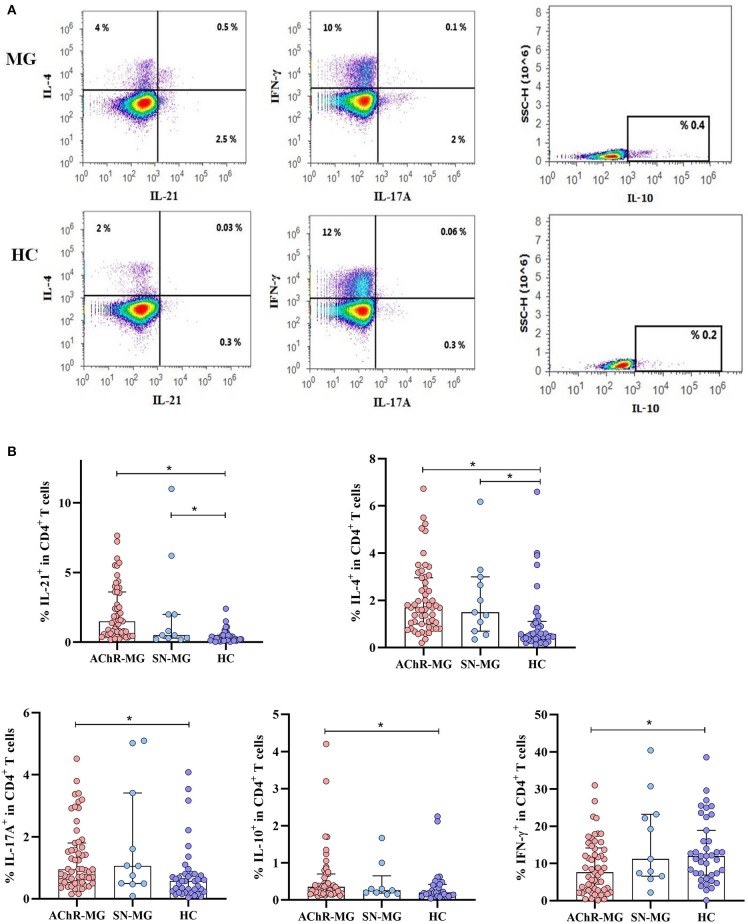
Cytokine production of CD4^+^ T cells in myasthenia gravis (MG) subgroups. **(A)** Measurement of intracellular IL-21, IL-4, IL-17A, IL-10, and IFN-γ in CD4^+^ T cells of a patient and a healthy control (HC) by flow cytometry after 4 h of stimulation with phorbol 12-myristate 13-acetate and ionomycin in cell culture. **(B)** The AChR-MG (*n* = 54) patients had higher IL-21, IL-4, IL-17A, and IL-10 (*p* < 0.001, *p* < 0.001, *p* = 0.001, and *p* = 0.014) and lower IFN-γ (*p* = 0.023) in CD4^+^ T cells compared to HC (*n* = 38). In the SN-MG group (*n* = 11), IL-21 (*p* = 0.032), and IL-4 (*p* = 0.010) were increased compared with those of HC. The results are compared with non-parametric tests (Kruskal–Wallis and Mann–Whitney *U*). * depicts a significant difference.

To understand the differential role of cytokines in AChR-MG and SN-MG development, IL-21, IL-4, IL-17A, IFN-γ, and IL-10 productions of CD4^+^ T cells were analyzed in the PBMCs *ex vivo*. The intracellular staining of CD4^+^ T cells is presented in Figure 1A. IL-21, IL-4, IL-17A, and IL-10 productions were increased in AChR-MG patients (*n* = 54, *p* < 0.001, *p* < 0.001, *p* = 0.001, and *p* = 0.014, respectively), whereas IFN-γ was lower compared to that in HC (*n* = 38, *p* = 0.023) (Figure 1B). IL-21 and IL-4 were also increased in SN-MG patients (*n* = 11) (*p* = 0.032 and *p* = 0.010). IL-17A and IL-10 were also slightly higher than the controls but without significance. There were no significant differences in the cytokine profile between AChR-MG and SN-MG patients. Cytokine productions were also not different between AChR-EO and AChR-LO MG subgroups, patients with or without thymectomy, or according to the thymic pathologies (data not shown). However, both in AChR-MG and SN-MG groups, but not in HC, women produced higher IFN-γ than men (12.3 vs. 5.5%, *p* = 0.008 and 19.3 vs. 3.0%, *p* = 0.034; data not shown).

### Effects of IS Treatment on Cytokines

**Figure 2:**
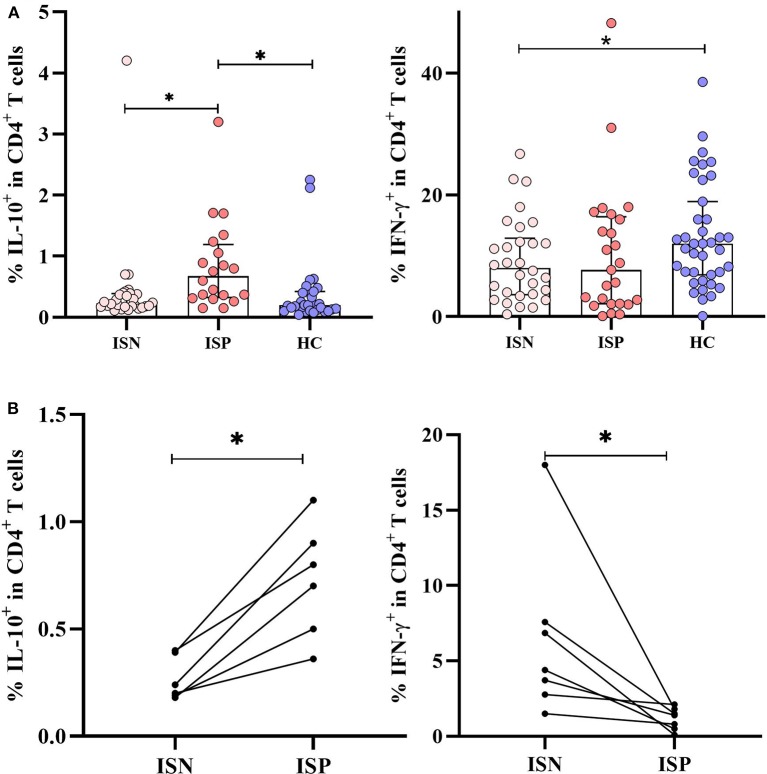
Effect of immunosuppressive (IS) treatment in myasthenia gravis patients with AChR antibodies (AChR-MG) patients. **(A)** IL-10 production was increased in IS-positive patients (*n* = 25) compared to that of IS-negative patients (*n* = 29) and healthy control (HC) (both *p* = 0.001). The patients who were not receiving IS treatment had lower IFN-γ^+^ CD4^+^ T cells compared to HC (*p* = 0.037). **(B)** The effect of IS treatment in sequential measurements of seven patients: The initial IL-10 and IFN-γ values of AChR-MG patients and the values after IS treatment are shown (*p* = 0.028 and *p* = 0.018). The results are compared with non-parametric tests (Kruskal–Wallis and Mann–Whitney *U*). * depicts a significant difference.

Cytokine production is influenced by IS treatment according to several studies (31, 32). This effect was evaluated only in AChR-MG patients separated as ISP (*n* = 25) or ISN (*n* = 29). IS treatment had an increasing effect on IL-10 production in ISP patients compared to that in HC as well as to the ISN patient group (both *p* = 0.001). IFN-γ production in ISN patients was lower than in HC (*p* = 0.037) (Figure 2A). The IFN-γ levels of ISP patients were similar to the ISN group and also relatively lower than those in the HC group. No significant effect of IS treatment on increased IL-21, IL-4, and IL-17A productions was observed (data not shown).

With another approach, seven AChR-MG patients who were initially not on IS treatment have been followed up to 6 months after starting the treatment. Measurements of the cytokines were performed in the same patients before and after the treatment. IL-10 increased in the CD4^+^ T cells of all patients on IS treatment (*p* = 0.028), supporting the above finding of treatment. On the contrary, IFN-γ decreased with the effect of IS treatment (*p* = 0.018) (Figure 2B). No other effect of IS treatment on cytokines was shown (data not shown). In these seven patients, five had improved clinically with reduced symptoms. As only two out of 11 patients in the SN-MG group were on IS treatment, this effect was not evaluated in this group.

### CD4^+^ T Cells and T Helper Subsets in MG Patients

**Figure 3:**
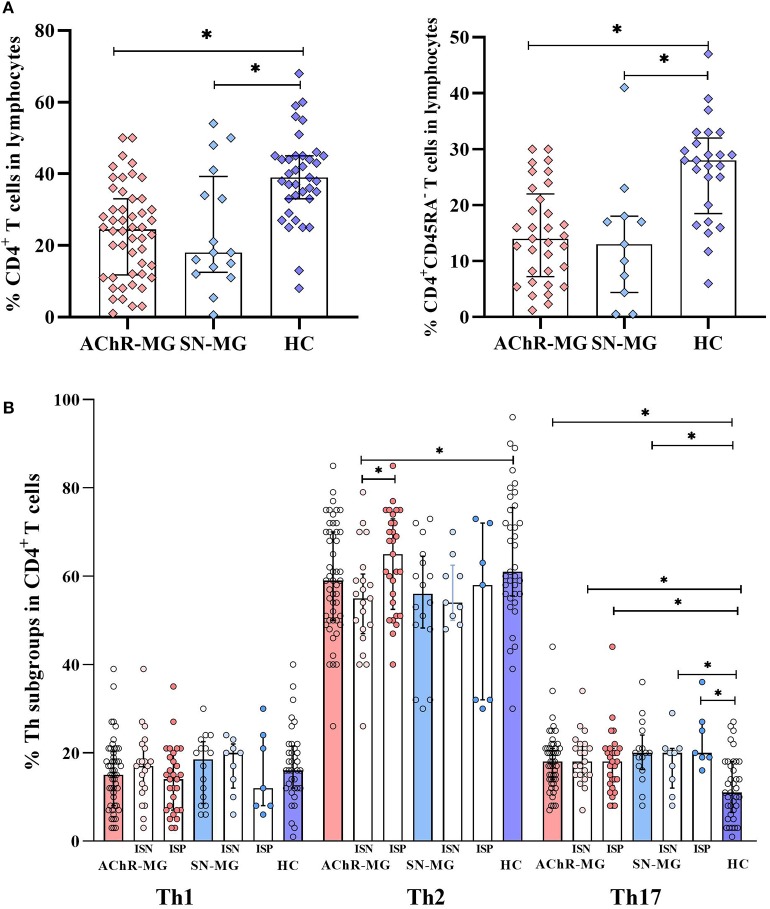
Distribution of CD4^+^ T cells and T helper subsets in the study groups. **(A)** CD4^+^ T and CD4^+^CD45RA^−^ T cells were lower in myasthenia gravis patients with AChR antibodies (AChR-MG) (*n* = 47, *n* = 30, both *p* < 0.001) and in myasthenia gravis patients without detectable antibodies (SN-MG) (*n* = 16, *p* = 0.004 and *n* = 11, *p* = 0.003) compared with those in HC (*n* = 37 and *n* = 25). **(B)** The Th17 (CXCR3^−^CCR6^+^) cell subset was increased in AChR-MG (*p* < 0.001) and SN-MG (*p* = 0.001) groups compared with that in HC. Both AChR-MG immunosuppressive (IS)-positive (*n* = 26) and IS-negative (*n* = 21) groups had higher Th17 cells compared to HC (*p* = 0.002 and *p* = 0.001). Immunosuppressive (IS) treatment induced a relative increase of Th2 cells (*p* = 0.021) which were significantly lower than those in HC (*p* = 0.013). Both IS-positive (*n* = 7) and IS-negative (*n* = 9) patients had higher Th17 cell populations (*p* = 0.001 and *p* = 0.036) in the SN-MG group. The results are compared with non-parametric tests (Mann–Whitney *U* test). * depicts a significant difference.

The findings of increased cytokines in AChR-MG and SN-MG lead us to identify the CD4^+^ T cells further and to characterize the cytokine producers in the blood. Among the lymphocytes, the CD4^+^ T cells were significantly decreased in both AChR-MG (*n* = 47, *p* < 0.001) and SN-MG (*n* = 16, *p* = 0.004) groups compared to HC group (*n* = 37). These decreases were also observed in the CD4^+^ T cells with the memory phenotype (CD45RA^−^) in AChR-MG (*n* = 30, *p* < 0.001) and SN-MG (*n* = 11, *p* = 0.003) groups compared with HC group (*n* = 25) (Figure 3A). We further characterized these CD4^+^ T cells in patients for their differentiation states into Th subgroups related to cytokine production. The Th subsets were identified by their differential expression of CXCR3 and CCR6 on CD4^+^ T cells; the proportions of Th1: CXCR3^+^CCR6^−^, Th2: CXCR3^−^CCR6^−^, and Th17: CXCR3^−^CCR6^+^ were measured among the CD4^+^ T cells (Supplementary Figure 1). When the distribution of the Th cell subsets was compared with HC, the Th17 cell population was increased both in AChR-MG and SN-MG patients (*p* < 0.001 and *p* = 0.001) (Figure 3B). Disease-onset age (EO vs. LO) as well as gender did not have any effect on the CD4^+^ T cell subset distribution. In 32% of the AChR-MG group who had been thymectomized, cell distribution was also not different from that of the non-thymectomized patients (data not shown).

### Effect of IS Treatment on Th Cells

As the cytokine production was affected by the IS treatment, similar effects were investigated in the Th cell subtypes. IS treatment did not change the observed increase of Th17 subset in AChR-MG patients. Both ISP (*n* = 26) and ISN (*n* = 21) groups had higher Th17 cells compared to HC (*n* = 37, *p* = 0.001 and *p* = 0.002, respectively). However, IS treatment induced a relative increase of Th2 cells (*p* = 0.021) which were significantly lower in the untreated group than in HC (*p* = 0.013). Similar to AChR-MG, both ISP (*n* = 7) and ISN (*n* = 9) groups had higher Th17 cell populations (*p* = 0.001 and *p* = 0.036) in SN-MG (Figure 3B).

### PD-1 Expression Was Higher on CD4^+^ T Cells in MG

**Figure 4:**
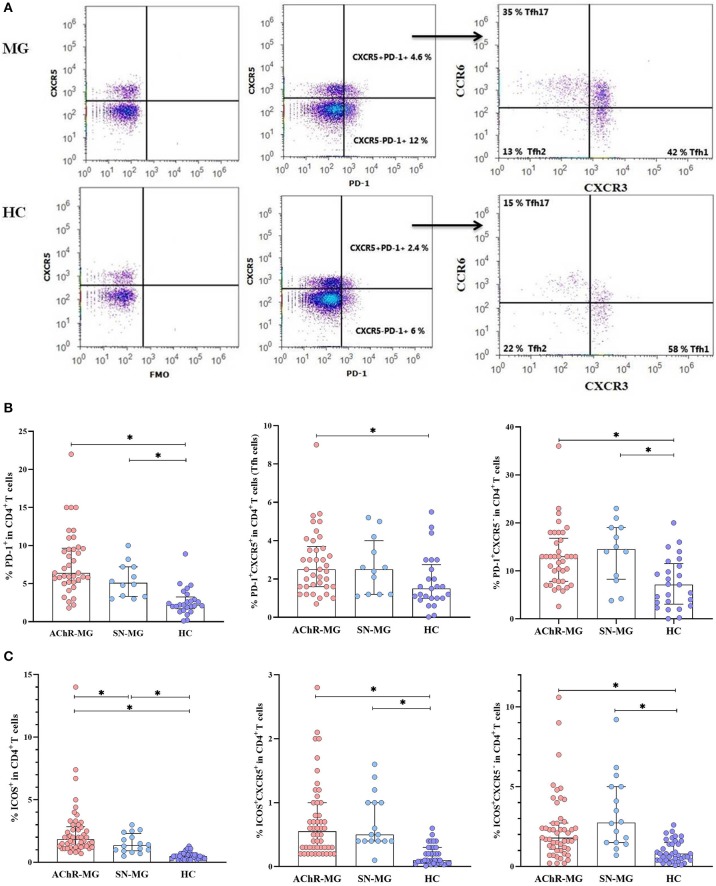
Increase of PD-1 and ICOS expression on the CD4^+^ T cells of myasthenia gravis (MG) patients. **(A)** The gating strategy to identify PD-1^+^CXCR5^+^ (cTfh) and PD-1^+^CXCR5^−^ populations in CD4^+^ T cells and cTfh subsets by flow cytometry is shown. **(B)** The frequencies of PD-1^+^ cells in CD4^+^ T cells were higher in MG patients with AChR antibodies (AChR-MG) (*n* = 35) and in MG patients without detectable antibodies (SN-MG) (*n* = 12) compared with those of HC (*n* = 25) (both *p* < 0.001). Both PD-1^+^CXCR5^+^ (cTfh) and PD-1^+^CXCR5^−^ populations in CD4^+^ T cells were higher in AChR-MG patients (*p* = 0.004 and *p* = 0.001), whereas PD-1^+^CXCR5^−^ in CD4^+^ T cells were increased in SN-MG (*p* = 0.006) compared with those in HC. **(C)** The proportions of ICOS^+^ cells in CD4^+^ T cells were increased both in AChR-MG (*n* = 47) and in SN-MG (*n* = 16) patients compared with those in HC (*n* = 37, both *p* < 0.001), including CXCR5^+^ (both *p* < 0.001) and CXCR5^−^ (both *p* < 0.001) subgroups of CD4^+^ T cells. The ICOS^+^ CD4^+^ T cells were lower in SN-MG than in AChR-MG (*p* = 0.05). The results are compared with non-parametric tests (Mann–Whitney *U* test). * depicts a significant difference.

In AChR-MG, higher productions of IL-21, IL-4, and also IL-17A were detected in total CD4^+^ T cells. Among the CD4^+^ T cells, the Tfh cells are considered as the major cell type for IL-21 and IL-4 productions (12, 13). The involvement of the cTfh cells in AChR-MG has also been reported (22, 23). To evaluate the functional state of the CD4^+^ T cells and their relatedness with the cTfh cells, firstly PD-1 expression was examined on CD4^+^ T cells with or without CXCR5 molecules (Figure 4A). We analyzed the blood cells of 35 AChR-MG and 12 SN-MG patients and 25 HC by flow cytometry. PD-1 expressing CD4^+^ T cells was higher in AChR-MG as well as in SN-MG patients compared to HC (both *p* < 0.001). Both PD-1^+^CXCR5^+^ (cTfh cells) and PD-1^+^CXCR5^−^ populations in CD4^+^ T cells were increased in AChR-MG patients (*p* = 0.004 and *p* = 0.001, respectively) compared with HC. Only the PD-1^+^CXCR5^−^ population in CD4^+^ T cells was significantly higher in SN-MG patients (*p* = 0.006, Figure 4B).

### ICOS Expression of CD4^+^ T Cell Was Increased in MG

ICOS and PD-1 are two molecules closely related to the function of Tfh cells. Previous studies have reported that ICOS and PD-1 are highly expressed on CD4^+^CXCR5^+^ T cells in the PBMCs of AChR-MG patients (22, 23, 36). As PD-1 was increased on total CD4^+^ T cells, covering CD4^+^CXCR5^+^ and CD4^+^CXCR5^−^ T cells, we analyzed ICOS expression also in these subpopulations separately in AChR-MG, SN-MG, and HC groups (Supplementary Figure 2). ICOS was increased on CD4^+^ T cells in both disease subgroups (both *p* < 0.001) while being lower in SN-MG than in AChR-MG (*p* = 0.05, Figure 4C). The ICOS^+^CXCR5^+^ and ICOS^+^CXCR5^−^ subpopulations were both higher in AChR-MG (both *p* < 0.001) and SN-MG patients (both *p* < 0.001, Figure 4C). No differences were observed between disease onset and other subgroups, with ICOS being higher in all patients (data not shown).

### cTfh Cells

Defining the PD-1^+^CXCR5^+^ cells among CD4^+^ T cells as cTfh cells (19, 33), we detected both PD-1 and CXCR5 molecules on CD4^+^ T cells. The gating strategy of PD-1^+^CXCR5^−^ and PD-1^+^CXCR5^+^ (cTfh) cells in CD4^+^ T cells and further cTfh subsets, such as Tfh1, Tfh2, and Tfh17, is shown in Figure 4A. To see a possible dominance of a cTfh subset in the increased population, we analyzed cTfh (PD-1^+^CXCR5^+^) cells by dividing these into subsets as cTfh1 (CXCR3^+^CCR6^−^), cTfh2 (CXCR3^−^CCR6^−^), and cTfh17 (CXCR3^−^CCR6^+^). The proportions of Tfh subsets did not differ in patients with AChR-MG and with SN-MG compared to HC (Supplementary Figure 3). The same comparisons performed in a smaller sample group on CD4^+^CD45RA^−^ memory T cell populations provided the same results (data not shown). No differences were observed between disease onset, thymectomy, or gender subgroups either.

### Effect of IS Treatment on PD-1^+^, ICOS^+^ CD4^+^ T Cells, or Tfh Cells

A possible role of IS treatment also on the disease-relevant CD4^+^ T cell populations has been evaluated by comparisons of PD-1, ICOS, and CXCR5 among CD4^+^ T cells. Although PD-1 positivity on CD4^+^ T cells was not affected significantly from IS treatment, PD-1^+^CXCR5^+^cells (cTfh cells) were higher in the untreated than in the treated patients (*p* = 0.012) and the HC (*p* = 0.001) in AChR-MG. A similar observation is also made in SN-MG: the untreated patients revealed an increase in PD-1^+^CXCR5^+^cells (cTfh cells) compared with the treated (*p* = 0.008) and HC (*p* = 0.007) groups.

**Figure 5:**
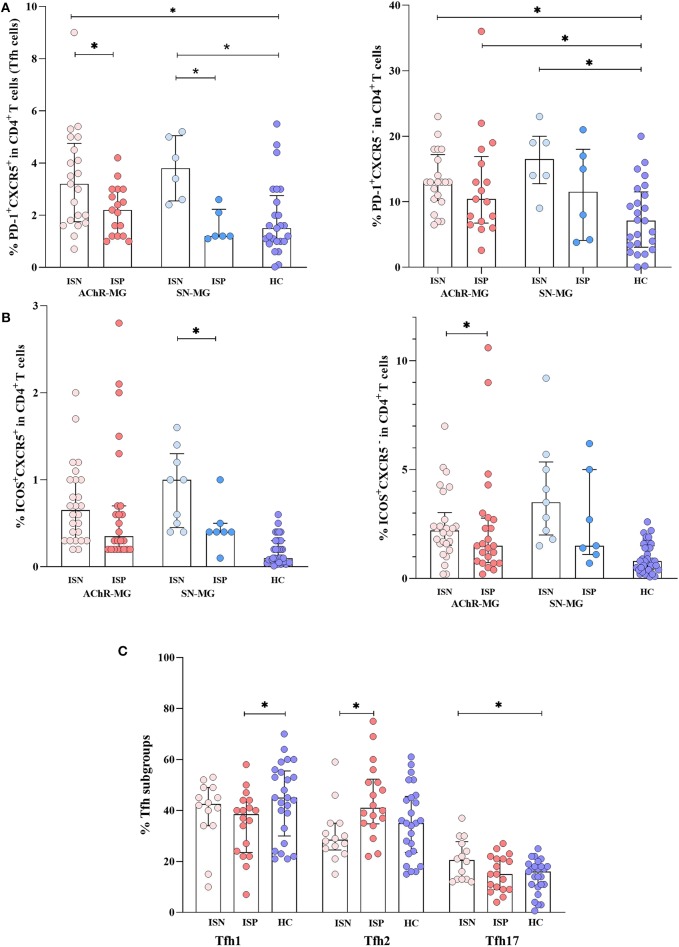
The effect of immunosuppressive treatment on T cell subpopulations. **(A)** The PD-1^+^CXCR5^+^cells (cTfh cells) were higher in untreated patients of both myasthenia gravis (MG) patients with AChR antibodies (AChR-MG) and MG patients without detectable antibodies (SN-MG) groups compared with those in HC (*p* = 0.001 and *p* = 0.007). The immunosuppressive (IS) treatment decreased the cTfh populations in both AChR-MG and SN-MG patients (*p* = 0.012 and *p* = 0.008). The PD-1^+^CXCR5^−^ cell population was higher in the IS-negative (ISN) subgroups of both AChR-MG and SN-MG patients compared to that in HC (*p* = 0.001 and *p* = 0.005). The PD-1^+^CXCR5^−^ cell population remained significantly higher than that of HC in the IS-positive (ISP) group of AChR-MG (*p* = 0.024). **(B)** Independent of CXCR5, the ICOS^+^ cells were higher in all subgroups. [These significant p values are not shown on the graph: ISN (*p* < 0.001) and ISP (*p* = 0.007) groups of AChR-MG and ISN (*p* < 0.001) and ISP (*p* = 0.012) groups of SN-MG for ICOS^+^CXCR5^−^ and ISN (*p* < 0.001) and ISP (*p* < 0.001) groups of AChR-MG and ISN (*p* < 0.001) and ISP (*p* = 0.003) groups of SN-MG for ICOS^+^CXCR5^+^)]. The ICOS^+^ on CXCR5^−^ cells in AChR-MG (*p* = 0.037) were higher, whereas on CXCR5^+^ cells in SN-MG (*p* = 0.029) these were reduced with treatment. **(C)** An increase of Tfh2 (*p* = 0.005) was induced by treatment compared to that in untreated patients. Higher Tfh17 cells (*p* = 0.036) in untreated patients and lower Tfh1 cells in treated patients were detected (*p* = 0.042) compared to those in HC. The results are compared with non-parametric tests (Mann–Whitney *U* test). * depicts a significant difference.

The PD-1^+^CXCR5^−^ cell population was higher in the ISN subgroups of both AChR-MG and SN-MG patients compared to HC (*p* = 0.001 and *p* = 0.005, respectively; Figure 5A). The PD-1^+^CXCR5^−^ cell population remained significantly higher than HC in the ISP group of AChR-MG (p = 0.024), while the higher levels in six SN-MG patients on treatment were not different (Figure 5A).

Independent of CXCR5, the ICOS^+^ cells were higher in the two disease groups with or without treatment compared with HC [ISN (*p* < 0.001) and ISP (*p* = 0.007) groups of AChR-MG, ISN (*p* < 0.001) and ISP (*p* = 0.012) groups of SN-MG for ICOS^+^CXCR5^−^, ISN (*p* < 0.001) and ISP (*p* < 0.001) groups of AChR-MG, and ISN (*p* < 0.001) and ISP (*p* = 0.003) groups of SN-MG for ICOS^+^CXCR5^+^)]. In AChR-MG ICOS^+^ on CXCR5^−^ cells (*p* = 0.037), while in SN-MG ICOS+ on CXCR5^+^ cells (*p* = 0.029) were reduced with treatment (Figure 5B).

The proportions of Tfh1, Tfh2, and Tfh17 cells have also changed with IS treatment in AChR-MG. An increase of Tfh2 (*p* = 0.005) was induced by treatment compared to the untreated patients (Figure 5C). Higher Tfh17 cells (*p* = 0.036) in untreated patients and lower Tfh1 cells in treated patients were detected (*p* = 0.042) compared to HC. These findings supported the effect of IS treatment shown on IL-10 and IFN-γ productions by CD4^+^ T cells in AChR-MG, whereas no differences were detected in SN-MG group (data not shown).

### CXCR5 and PD-1 Were Higher on Thymocytes in Thymus

**Figure 6:**
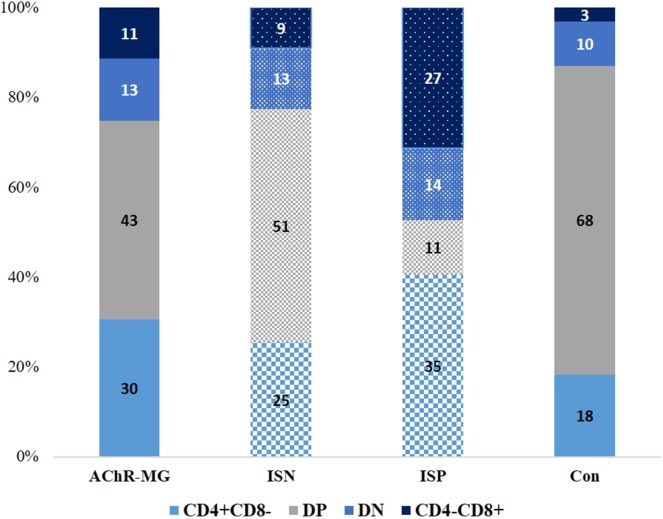
Proportional distribution of the maturational stages of thymocytes in different groups. CD4^+^CD8^−^, CD4^+^ CD8^+^ (double-positive, DP), CD4^−^CD8^−^ (double-negative, DN), and CD4^−^CD8^+^ thymocytes in 37 myasthenia gravis patients with AChR antibodies (AChR-MG) [16 immunosuppressive (IS)-treated patients, ISP, and 22 not IS-treated patients, ISN] and 20 control (Con) patients. Higher proportions of CD4 single-positive (CD4^+^CD8^−^) and DN thymocytes were observed in AChR-MG cases compared to those in the Con group (*p* = 0.015 and *p* = 0.007). The CD4 and CD8 DP thymocytes were decreased in the AChR-MG group (*p* = 0.002) in comparison to those in the control group. The proportions of CD4^+^CD8^−^ (*p* = 0.05) and DN (*p* = 0.001) thymocytes were higher and DP thymocytes (*p* < 0.001) were decreased when the patients were on IS treatment compared with patients without treatment at the time of thymectomy. Parametric tests are used for comparisons of subpopulations by using age as covariate.

Similar to the typical GC Tfh cells, cTfh with CD4^+^CXCR5^+^ phenotype are also considered as helpers of B cells in producing antibody *via* IL-21. In patients with thymic hyperplasia, the thymic tissue contains GC with similar organization to secondary or tertiary lymphoid tissues in MG (7). Moreover, similar changes of cytokines have been demonstrated in the thymus of patients (35). Along with the cTfh cells, we evaluated CXCR5, PD-1, or ICOS expression on thymocytes obtained from the thymic tissues to search for cells in the thymus resembling Tfh cells. However, as the maturational states of all thymocytes would not be the same, we firstly analyzed thymocytes for CD4 and CD8 expression patterns to characterize the cells. Thymocytes from 37 AChR-MG (33 hyperplasia and four involution cases) and 20 control thymi (Con) revealed changes in the maturational stages of the thymocytes in MG as reported before (37, 38) (Figure 6). Although the ages of the control tissue group varied considerably and revealed a significant difference from the ages of the patient group (*p* = 0.023), comparisons by controlling the effect of age also demonstrated significant differences between Con and AChR-MG tissue cells. In AChR-MG cases, higher proportions of CD4 single-positive (CD4^+^CD8^−^, SP, *p* = 0.015) and double-negative (DN, *p* = 0.007) thymocytes were observed compared to Con group. CD4 and CD8 double-positive (DP) thymocytes were decreased proportionally in AChR-MG group (*p* = 0.012). Comparisons revealed that the proportions of CD4^+^CD8^−^ SP (*p* = 0.05) and DN (*p* = 0.001) thymocytes were even higher and DP thymocytes (*p* < 0.001) were lower in patients on IS treatment at the time of thymectomy compared with samples from IS-naïve patients (Figure 6).

**Figure 7:**
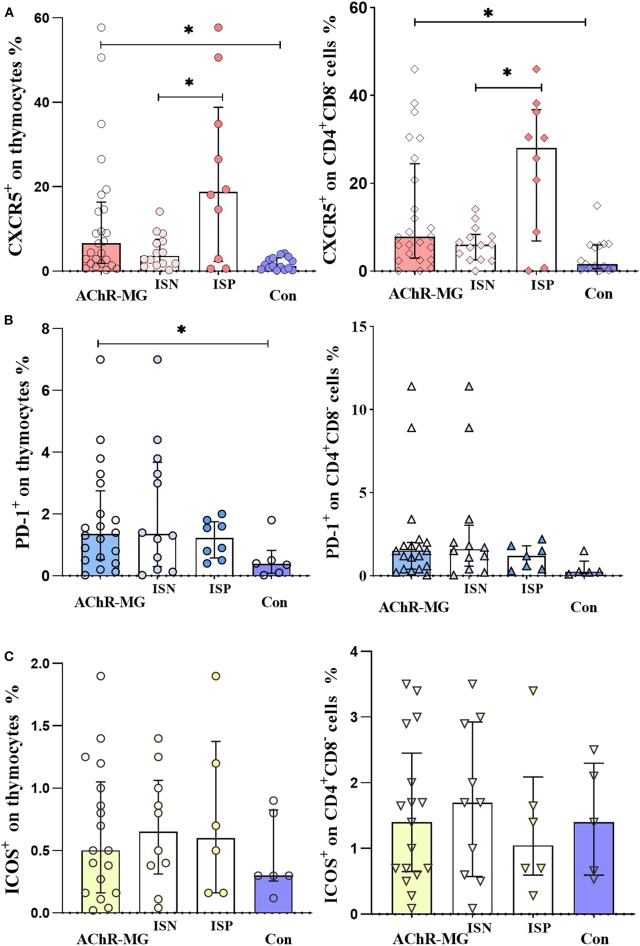
CXCR5, PD-1, and ICOS expression on thymocytes. **(A)** CXCR5 increase on thymocytes of myasthenia gravis patients with AChR antibodies (AChR-MG) (*n* = 24) compared with control thymi (Con) (*n* = 14) (*p* = 0.002). Increase of CXCR5 on CD4^+^ CD8^−^ single-positive thymocyte population of AChR-MG patients compared with Con (*p* = 0.004). The proportions in IS-treated patients (ISP) were higher than in untreated patients (ISN) group (*p* = 0.016) and Con (*p* = 0.007), with the ISN group being higher than Con (*p* = 0.042) as well. **(B)** The PD-1 on thymocytes (*p* = 0.039) and on CD4^+^ CD8^−^ cells (*p* = 0.028) of AChR-MG patients (*n* = 18) were higher than those of Con (*n* = 5). **(C)** ICOS expression on thymocytes and on CD4^+^ CD8^−^ thymic cells. Non-parametric tests (Mann–Whitney *U* test) were used for comparisons between groups. * depicts a significant difference.

An increase of CXCR5^+^ thymocytes in the thymi of AChR-MG patients was observed compared to Con tissues (*p* = 0.002) (Figure 7). The thymic samples from patients with or without IS treatment at the time of thymectomy (10 ISP and 14 ISN patients) revealed an increase in the treated patients compared to the untreated ones (*p* = 0.033) and Con group (*p* = 0.007), although the ISN patients also had higher CXCR5 on thymocytes (*p* = 0.023). The thymocyte subpopulation that expresses higher CXCR5 has been identified as the more mature CD4^+^CD8^−^SP thymocytes in AChR-MG compared to Con (*p* = 0.004, Figure 7). In IS treatment-receiving patients, CXCR5^+^ cells in CD4^+^CD8^−^ SP population were higher than ISN group (*p* = 0.016) and Con (*p* = 0.007). However, these cells were also higher in ISN group compared with Con (*p* = 0.042).

When thymocytes were stained for PD-1 and ICOS expression, an increase of PD-1 was detected on the thymocytes and the CD4^+^CD8^−^ SP cells of AChR-MG patients (seven ISP and 11 ISN patients) compared to Con (*p* = 0.039 and *p* = 0.028, respectively) (Figure 7). The expression of ICOS did not show any significant differences in the groups.

In a small number of samples (*n* = 5), we also compared CD4^+^CXCR5^+^ cells between the thymus and the blood of MG patients. In this group, all patients had hyperplastic thymus and three of these patients were on IS treatment at the time of thymectomy when the PBMCs and thymic cells were obtained. The CD4^+^CXCR5^+^ population was higher in blood compared with thymus (11.8 vs. 0.5%). The distribution of chemokine receptors on CD4^+^CXCR5^+^ populations revealed lower Tfh2 (44 vs. 81%) and higher Tfh17 (36 vs. 10%) cell proportions in the blood compared to thymus in this small sample group (Supplementary Figure 4).

## Discussion

Being a prototypic autoimmune disease mediated by auto-antibodies, MG and its subtypes have been the subject of investigations on the interaction of T and B cells. In a previous study, we had shown increased IL-17A, IFN-γ, and IL-21 production in mainly MuSK-MG patients, whereas the AChR-MG patients, in comparison, did not reveal any differential cytokine production in response to non-specific T cell stimulation (26). In the present study, we demonstrate increased IL-4, in addition to IL-21 and IL-17A productions of CD4^+^ T cells from IS treated as well as untreated AChR-MG patients. Although IL-4, IL-21, and IL-17A have pointed at the impact of cTfh cells, the cytokine-producing and *in vivo*-activated cells were identified not necessarily as cTfh cells but had surface markers such as PD-1 or ICOS with or without CXCR5. Findings in the thymic samples from AChR-MG patients revealed an IS treatment-related increase of CXCR5 as well. The results obtained from a smaller sample group of SN-MG patients provided support for their common immunological features with AChR-MG. The complex cytokine network of regulation for auto-antibody production has been elucidated further in this study.

### Cytokines in MG

The dysregulation of cytokines from Th1, Th17, and Tfh populations is associated with the pathogenesis of many autoimmune diseases (39). Imbalances of related cytokines are reported in MG with differences between studies. Higher levels of IL-21 and IL-6 (23) were not confirmed in the sera of AChR-MG patients in other studies (26, 29). Increased IL-17 (27, 28, 40) was not observed in other studies in the sera or the culture supernatants of AChR-MG patients. Variations in these measurements were reduced by cellular evaluation of the cytokines. The production of both IFN-γ and IL-17 and the absence of IL-10 expression in response to AChR of the CCR6^+^ memory T cells in MG indicated a pro-inflammatory pathogenic phenotype with a recent approach (30). Thymic studies also supported the importance of IL-17, IFN-γ, IL-21, and TNF-α and related effects in MG (35, 41). The importance of IL-21 in MG has been shown to be associated with activated Tfh1 and Tfh17 cells being their major product (23). High frequencies of cTfh-Th17 cells with increased IL-21 mRNA expression confirmed this finding (36). The present study evaluated both Th- and Tfh-related cytokines. Th17-related activity and the relevance of IL-17 and IL-21 have also been demonstrated in MuSK-MG patients (26, 42). The findings of this study confirm the importance of IL-17 as a Th17-related cytokine and IL-21 as a Tfh cytokine *ex vivo* without stimulation in a relatively big sample. In addition to IL-17A and IL-21, a robust increase of IL-4 production in the CD4^+^ T cells also provides evidence that the cytokine activity from AChR-MG patients exhibits a “Th17/Tfh” signature.

### Tfh or Other CD4^+^ T Cell Types

Tolerance breakdown during B cell development (43) and aberrant selection in GC can increase the production of autoantibodies (44). Tfh cells are major immune regulators in B cell activation, differentiation into plasma cells, and antibody production in the lymphoid tissues (14). Isotype switching to IgG subclasses as in many autoimmune responses including MG is also subject to regulation by Tfh cells. With the observation of cTfh cells in the blood, several reports are published on the role of these cells in autoimmune diseases such as RA (17), SSc (19), and MG (23, 45). In MG, an increase of PD-1^hi^CXCR5^+^CD4^+^ or ICOS^hi^CXCR5^+^CD4^+^ T cell populations and a correlation with AChR antibodies were shown (22). Considering the ICOS expression of CXCR5^+^CD4^+^ cells as activation of cTfh, their higher IL-21 have also been demonstrated in AChR-MG (23). With a similar approach to the present study, when the cTfh cells are subtyped by receptors, the increased group has been determined as the cTfh–Th17 cells in the blood (36). The present results also confirmed the increase of cTfh cells defined as PD-1^+^CXCR5^+^CD4^+^ T cells. ICOS^+^CXCR5^+^CD4^+^ T cells were also increased in the present study group of two different disease subtypes. However, the observation of the parallel increase of the CXCR5^−^CD4^+^ T cells as ICOS^+^CD4^+^ and PD-1^+^CD4^+^ populations in both AChR-MG and SN-MG patients raised the possibility that another subtype of T cells can be involved in MG for this cytokine imbalance. The Tph cell group shown in the synovium of RA patients provided evidence for a new cell group with helper function to antibody production induced at the peripheral tissue (20). The peripheral or even thymic presence of PD-1- or ICOS-expressing cells may indicate similarly effective cell types in MG.

On the other hand, when the functional effects of cTfh cells in the blood of patients with SSc were compared, the positive correlation with plasma cell differentiation and the increase of IgG levels in co-cultures with B cells were mainly observed with PD-1^+^CXCR5^+^, but less with PD-1^+^CXCR5^−^CD4^+^ T cells. Both PD-1^+^CXCR5^+^ and PD-1^+^ CXCR5^−^ CD4^+^T cells have been shown to produce high IL-21 (19). Although Tfh cells defined as CD4^+^CXCR5^+^ T cells appear to be responsible for antibody production in lymphoid tissues, the data suggest that PD-1 expression on CD4^+^ T cells may be more important in the interaction of these cells with B cells. Both increases of ICOS and PD-1 expression on CD4^+^ may contribute to the peripheral regulation of autoantibody production in AChR-MG. Further functional comparisons of these cell populations are needed to elucidate their effect in disease development.

The SN-MG patients revealed similarities to the AChR-MG patients. Although CD4^+^CXCR5^+^PD-1^+^ (cTfh) cells were increased only in AChR-MG, both PD-1^+^CD4^+^ and ICOS^+^CD4^+^ T cell populations were higher in AChR-MG as well as in SN-MG. The Th17 population was increased in both disease subgroups compared to the HC. These findings suggest that AChR-MG and SN-MG patient groups, which are clinically similar to each other, may have a similar profile of cytokine regulations with some differences.

### IS Treatment Effect

In this study, changes induced by IS treatment in cytokine production and subsets of CD4^+^ T cells were detected by measurements of separate groups as well as in sequential measurements in a group of AChR-MG patients. To rule out the variability of cytokine data in MG studies which may be IS treatment-related, relatively big groups and *ex vivo* cellular samples were studied.

IS treatment induced an increase of IL-10 and a decrease of IFN-γ production by CD4^+^ T cells. In addition, Th2 and Tfh2 cell populations were increased in ISP patients, implicating an effect of IL-10 on the differentiation of Th2 and Tfh2 populations in AChR-MG. On the other hand, cTfh and cTfh1 populations were decreased in ISP AChR-MG patients, which may be related to the lower IFN-γ levels of ISP patients. A decrease of Tfh population with IS treatment has been documented in SLE (33) and also in IgG4-related disease (34). In a recent report, CD4^+^PD-1^+^ and CD4^+^ICOS^+^ T cells were decreased after IS treatment in MG as well (45). Soluble forms of PD-1 and ICOSL were also increased in untreated patients and affected by IS treatment. However, we did not observe decreases of PD-1 or ICOS expression on CD4^+^ T cells with IS treatment.

On the other hand, the treatment effects in SN-MG were not properly evaluated as the number of patients was not as high as those of the other groups and the distribution in the treatment groups was not balanced, which has been the main limitation of the present study.

### Thymic Changes Related to Peripheral Findings

In the thymic tissue of AChR-MG patients with hyperplasia, ectopic GC-like persistent structures are detected and evidences are presented that B cells are chemo-attracted and activated at these structures, resulting in antibody production (46). Co-localization of Tfh and B cell within the ectopic GC in MG thymus has also been reported (24). Evaluating the possible contribution of thymic pathology in autoantibody and cytokine production, we screened thymocytes for the relevant molecules. Preliminary observations provided support for the changes toward differentiation of Tfh cells in the thymic tissues of AChR-MG patients, mainly in more mature CD4 single-positive thymocytes and also related to IS treatment. However, functional evidence is lacking to prove this development. As previously reported, glucocorticoids induce atrophy in the thymus by acting on DP thymocytes which preferentially express the glucocorticoid receptors in the thymus (47). Locally produced, thymic endothelial cell-derived glucocorticoids in the thymus have also been shown to participate in antigen-specific thymocyte development (48, 49). In that sense, exogenous glucocorticoids may have an effect on antigen-specific T cell development and preferentially on the thymocyte development of a Tfh-like phenotype. As an alternative explanation, the active Tfh cells in the thymus may be insufficiently suppressed by glucocorticoids, leading to a persistent disease activity in MG, and may explain the favorable response to thymectomy.

## Conclusion

In conclusion, MG pathogenesis is associated with increased IL-21, IL-4, and IL-17A levels and higher frequencies of ICOS and PD-1 expressing CD4^+^ T cells. AChR-MG and SN-MG patients have similar cytokine productions and subsets of CD4^+^ T cell populations. Th2 or Tfh2 cells and IL-10 are mainly increased with the effect of IS treatment, which may have a compensating role. However, the production of IL-21, IL-17, and IL-4 are not affected by IS treatment. Blockade of T cell activation pathways of specific cell populations with distinctive surface markers which are not targeted by IS treatment will be a challenge for potential therapeutic approaches in MG.

## Data Availability Statement

The datasets generated for this study are available on request to the corresponding author.

## Ethics Statement

The studies involving human participants were reviewed and approved by Ethical Review Board of Istanbul Medical Faculty. The patients/participants provided their written informed consent to participate in this study.

## Author Contributions

MÇ, HD, YP, and GS-D contributed to the design of the study and to the planning of experiments. MÇ and GS-D wrote the manuscript. MC, HD, FA, BÖ, AÇ, GG, MM, MT, VY, OA, ÖD, YP, and GS-D were responsible for the ethical permission and for the clinical selection and diagnostic evaluation of patients and controls. MÇ, MH, and SY performed the experiments. MÇ and GS-D organized the figures and the tables.

## Conflict of Interest

The authors declare that the research was conducted in the absence of any commercial or financial relationships that could be construed as a potential conflict of interest.

## Supplementary Material

Supplementary Figure 1 Gating strategy to identify Th1 (CXCR3^+^CCR6^−^), Th2 (CXCR3^−^CCR6^−^) and Th17 (CXCR3^−^CCR6^+^) populations in CD4^+^ T cells by flow cytometry is shown.

Supplementary Figure 2 Gating strategy to identify ICOS^+^CXCR5^+^ (cTfh) and ICOS^+^CXCR5^−^ populations in CD4^+^ T cells by flow cytometry is shown.

Supplementary Figure 3 Tfh1(CXCR3^+^CCR6^−^) Tfh2 (CXCR3^−^CCR6^−^) and Tfh17 (CXCR3^−^CCR6^+^) cell subsets among CD4^+^CXCR5^+^ T cells in AChR-MG and SN-MG groups compared with HC.

Supplementary Figure 4 Thymic (t) and circulating (c) Tfh1(CXCR3^+^CCR6^−^), Tfh2 (CXCR3^−^CCR6^−^) and Tfh17 (CXCR3^−^CCR6^+^) cell subsets among CD4^+^CXCR5^+^ T cells detected in samples of five AChR-MG patients.
