# Supplementary figures and images for: CD4+ T Cells of Myasthenia Gravis Patients Are Characterized by Increased IL-21, IL-4, and IL-17A Productions and Higher Presence of PD-1 and ICOS
Source: Front Immunol. 2020 May 19;11:809. doi: 10.3389/fimmu.2020.00809 (PMC7248174; doi:10.3389/fimmu.2020.00809)

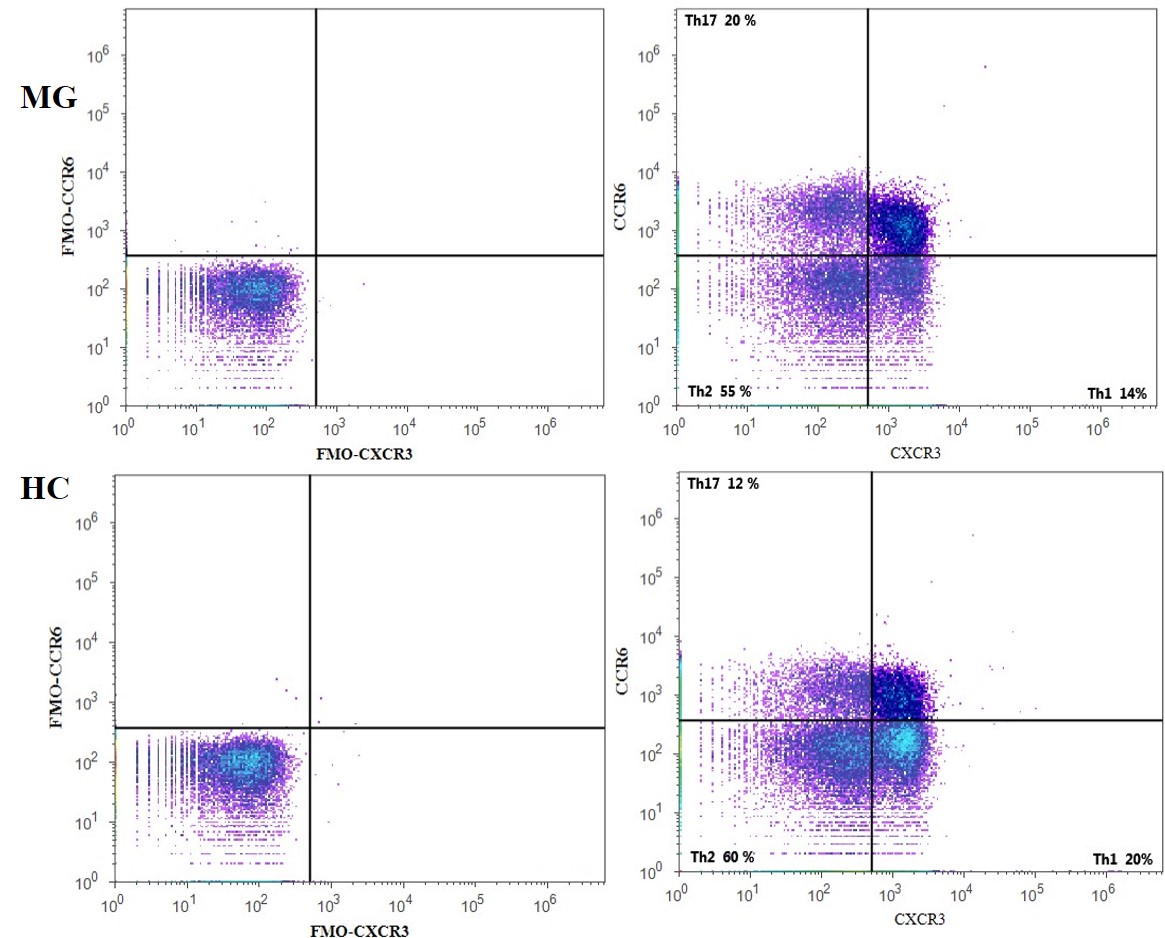

Supplement: Supplementary Figure 1 — Gating strategy to identify Th1 (CXCR3+CCR6−), Th2 (CXCR3−CCR6−) and Th17 (CXCR3−CCR6+) populations in CD4+ T cells by flow cytometry is shown. [file Image_1.JPEG]

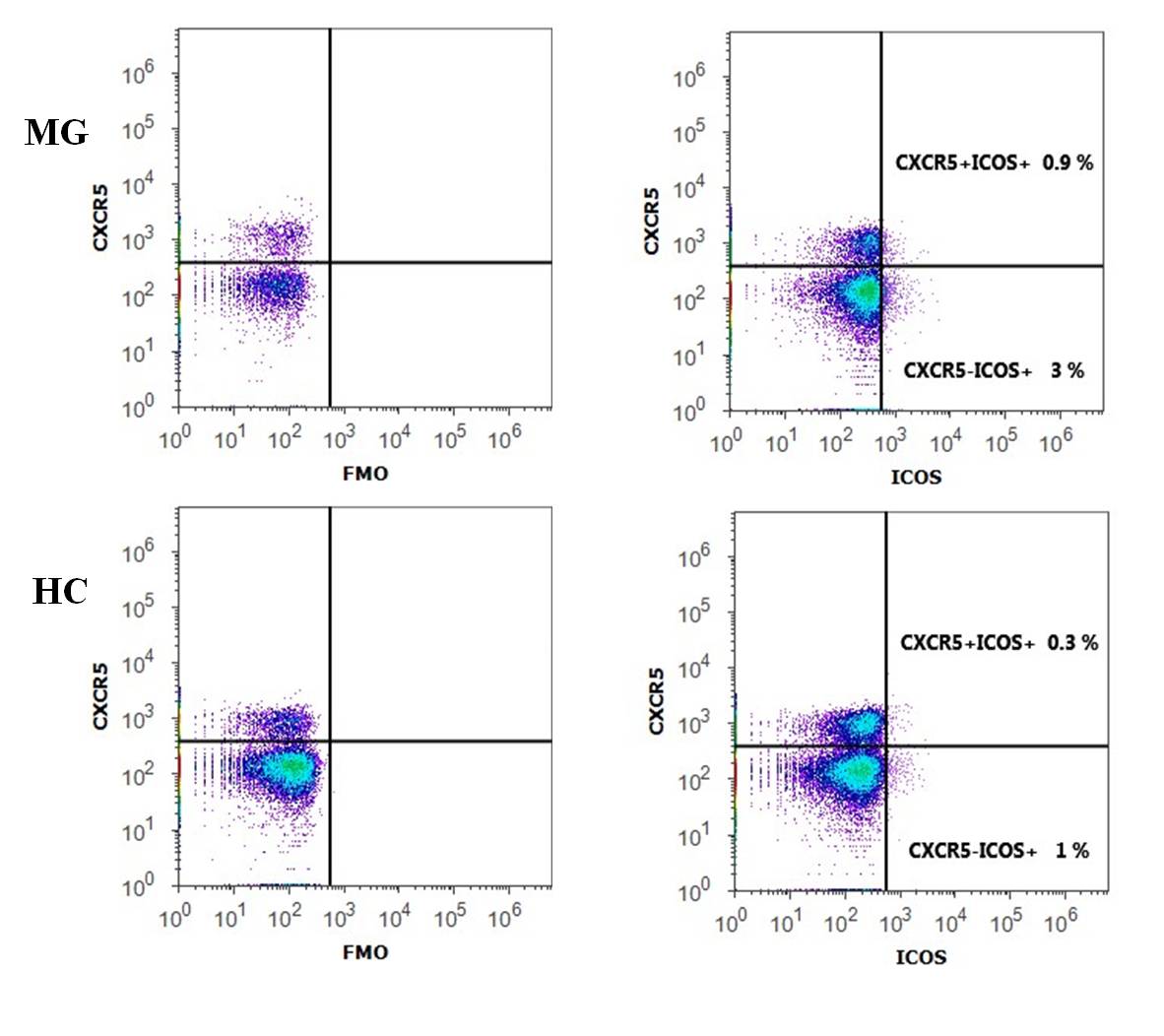

Supplement: Supplementary Figure 2 — Gating strategy to identify ICOS+CXCR5+ (cTfh) and ICOS+CXCR5− populations in CD4+ T cells by flow cytometry is shown. [file Image_2.JPEG]

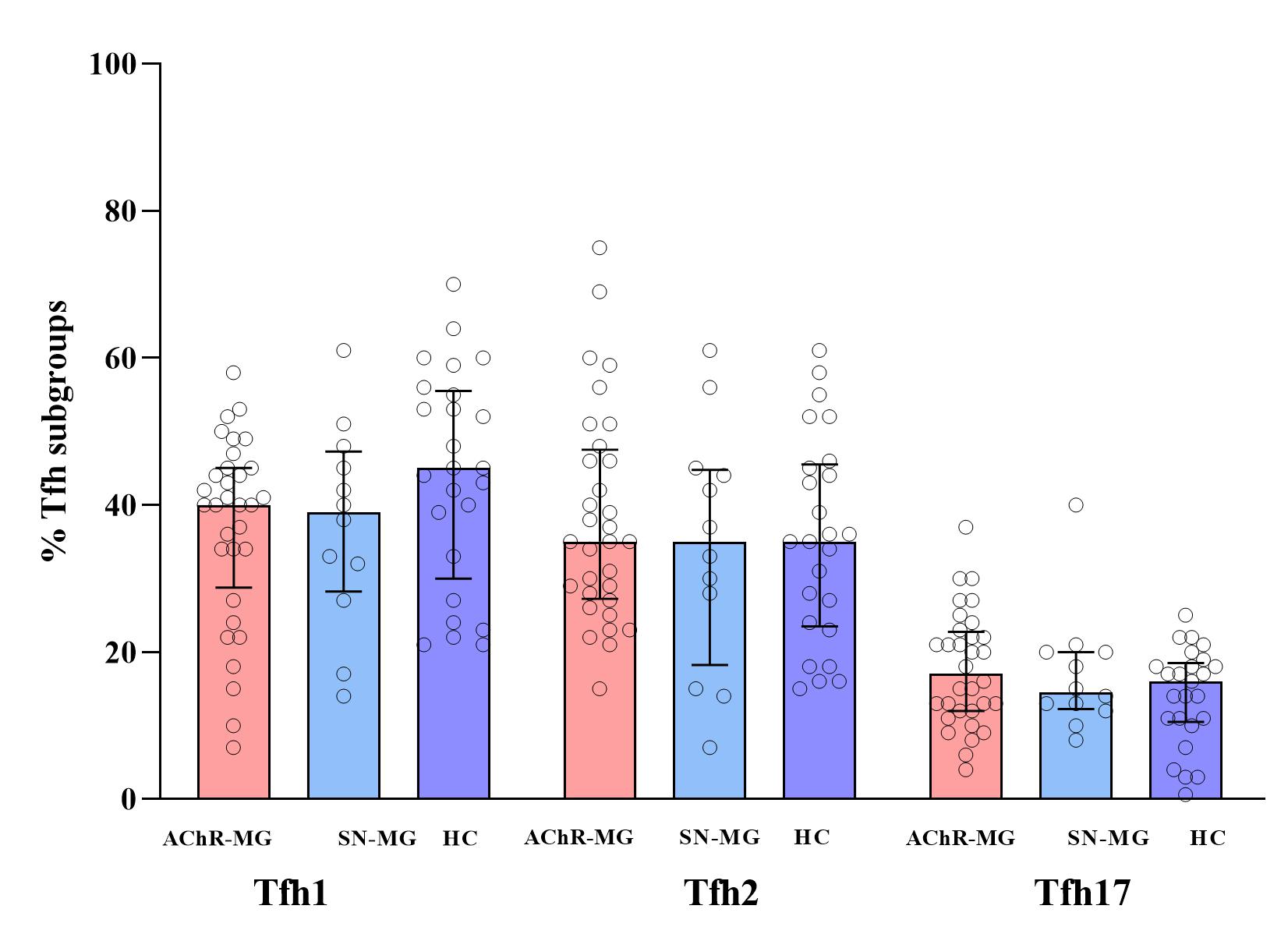

Supplement: Supplementary Figure 3 — Tfh1(CXCR3+CCR6−) Tfh2 (CXCR3−CCR6−) and Tfh17 (CXCR3−CCR6+) cell subsets among CD4+CXCR5+ T cells in AChR-MG and SN-MG groups compared with HC. [file Image_3.JPEG]

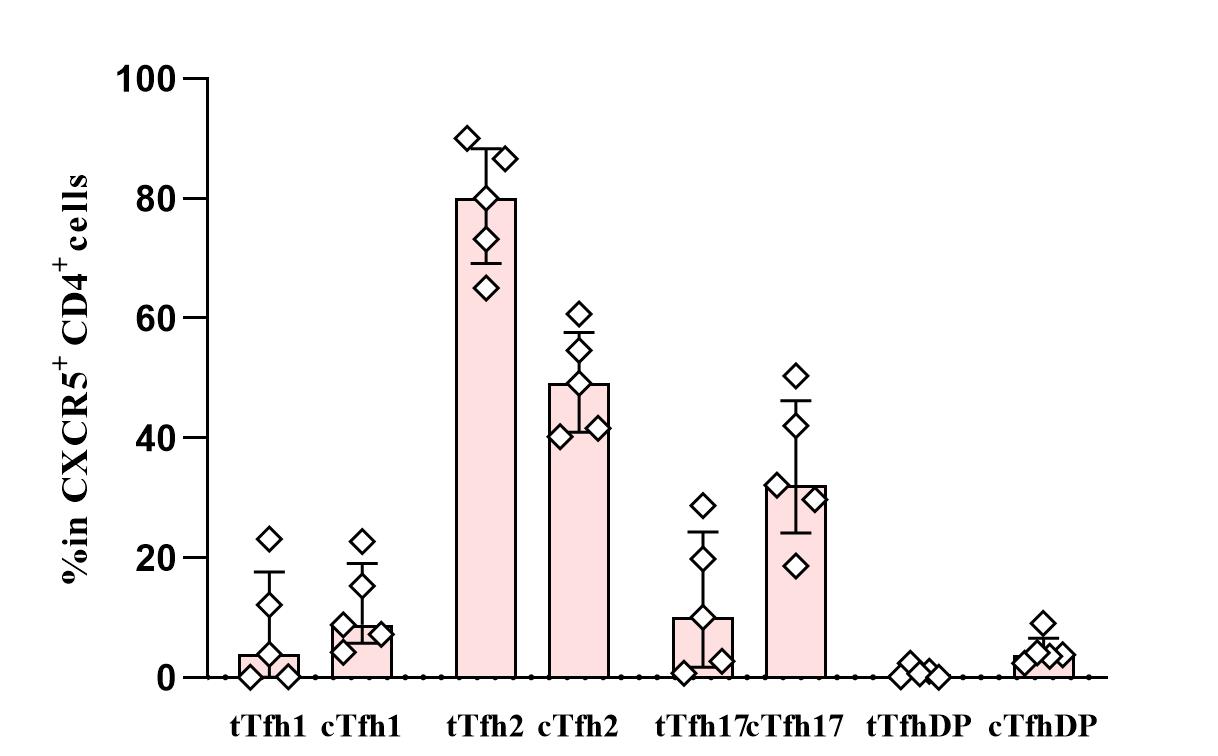

Supplement: Supplementary Figure 4 — Thymic (t) and circulating (c) Tfh1(CXCR3+CCR6−), Tfh2 (CXCR3−CCR6−) and Tfh17 (CXCR3−CCR6+) cell subsets among CD4+CXCR5+ T cells detected in samples of five AChR-MG patients. [file Image_4.JPEG]
